# A review of sustainable biodiesel production using biomass derived heterogeneous catalysts

**DOI:** 10.1002/elsc.202100025

**Published:** 2021-10-22

**Authors:** Semakula Maroa, Freddie Inambao

**Affiliations:** ^1^ College of Agriculture Science and Engineering Discipline of Mechanical Engineering Green Energy Group University of KwaZulu‐Natal Durban South Africa

**Keywords:** biomass waste, heterogeneous catalysts, high catalytic activity, homogeneous catalysts, transesterification reaction

## Abstract

The production of biodiesel through chemical production processes of transesterification reaction depends on suitable catalysts to hasten the chemical reactions. Therefore, the initial selection of catalysts is critical although it is also dependent on the quantity of free fatty acids in a given sample of oil. Earlier forms of biodiesel production processes relied on homogeneous catalysts, which have undesirable effects such as toxicity, high flammability, corrosion, by‐products such as soap and glycerol, and high wastewater. Heterogeneous catalysts overcome most of these problems. Recent developments involve novel approaches using biomass and bio‐waste resource derived heterogeneous catalysts. These catalysts are renewable, non‐toxic, reusable, offer high catalytic activity and stability in both acidic and base conditions, and show high tolerance properties to water. This review work critically reviews biomass‐based heterogeneous catalysts, especially those utilized in sustainable production of biofuel and biodiesel. This review examines the sustainability of these catalysts in literature in terms of small‐scale laboratory and industrial applications in large‐scale biodiesel and biofuel production. Furthermore, this work will critically review natural heterogeneous biomass waste and bio‐waste catalysts in relation to upcoming nanotechnologies. Finally, this work will review the gaps identified in the literature for heterogeneous catalysts derived from biomass and other biocatalysts with a view to identifying future prospects for heterogeneous catalysts.

**Abbreviations**
Al_2_O_3_
: aluminum oxide
Ba: barium
BaO: barium oxide
C=O: carbonyl group
Ca: calcium
Ca_10_ (PO_4_)_6_ (OH)_2_, HAP: hydroxyapatite
Ca_2_FeO_5_
: calcium ferrite oxide
CaCO_3_
: calcium carbonate
CaMg (CO_3_)2: dolomite
CaMnO_3_
: orthorhombic perovskite
CaO: calcium oxide
CaO‐CeO_2_
: cerium (IV) oxide
CaTiO_3_
: calcium titanate
CaZrO_3_
: calcium zirconate
CH_3_OK: potassium methoxide
CH_3_ONa: sodium methoxide
CO_2_
: carbon dioxide
COOH: carboxylic group
CsZrO_2_
: chitoson zirconium oxide
DNA: deoxyribonucleic acid
FAAE: fatty acid alkyl esters
FAME: fatty acid methyl ester
Fe_3_O_4_
: magnetic oxide (magnetite)
FeCl_3_
: ferric chloride
FFA: free fatty acid
FTIR: Fourier transform infrared
H_2_SO_4_
: sulfuric acid
H_3_PO_4_
: phosphoric acid
HCL: hydrochloric acid
HCs: hydrocarbons
HTHP: higher temperatures and pressure
K_2_CO_3_
: potassium carbonate
K_2_O: potassium oxide
KF: potassium fluoride
KI: potassium iodide
KNO_3_
: potassium nitrate
KOH: potassium hydroxide
La_2_O_3_
: lanthanum oxide
Li/ZnO: lithium (Li) doped zinc oxide
Mg: magnesium
MgO: magnesium oxide
MSW: municipal solid waste
NaOH: sodium hydroxide
NO_X_
: nitrogen oxide
OH: hydroxyl
PrSO_3_H‐SBA‐15: propylsulfonic acid
PSRD: pressure swing reactive distillation
RD: reactive distillation
SEM: scanning electro‐microscopy
SiO_2_
: silicon dioxide
SO_3_H: sulfonic acid
Sr: strontium
SRO: strontium oxide
TGA: thermogravimetric analysis
XRD: x‐ray diffraction
Zn (NO_3_)_2_
: zinc nitrate
ZnO: zinc oxide

## 1 INTRODUCTION

There has been increased interest in recent years in heterogeneous catalysts also known as solid catalysts for the production of biodiesel. The concerns regarding the increase in global warming and with growing environmental awareness and the depletion of fossil sources and fluctuating global oil prices there is a need to create diversification through alternative renewable energy sources such as biofuel and biodiesel [1, 2, 3]. At present global primary consumption by fossil fuel stands at 80% of which 58% is directly consumed in the transportation sector [4].

Production of biodiesel using lipase catalysis offers many benefits such as ease of product separation, reduction in wastewater treatment and elimination of side reactions [5, 6]. Since conventional homogeneous alkaline catalyzed reactions cannot achieve high free fatty acid content use of immobilized lipase makes it possible [7]. This directly offers an alternative to process FFA and FAAE (fatty acid alkyl esters) at mild reaction conditions. However, it is important to note that lipase have some disadvantages such as slow reaction and run a risk of enzyme deactivation due to methanol concentration [8].

This massive consumption of fossil fuel calls for alternative renewable energy sources. Sources commonly preferred include hydrogen, natural gas, syngas and biofuel. Biofuel is sustainable and environmentally friendly. The main players of the global biofuel industry are the United States of America, Brazil the European Union and the Asian countries [1, 9]. Biofuels and biodiesel add economic value to the feedstock chain by increasing job opportunities, income, taxes and investment in plant and equipment [10]. Uptake of alternative fuel and increased application in the transport sector will lessen dependence on crude oil imports. This supports domestic agriculture development by increasing domestic opportunities in the agricultural sector.

Alternative fuels offer more merits than demerits compared to fossil fuel resources. The merits include environmental benefits such as non‐toxicity, biodegradability, high‐energy efficiency, reduction in emission of CO_2_, sulfur, particulate emissions and NO_X_ [11, 12, 13]. It is estimated that biodiesel fuel yields 90 to 40% of the energy invested in its production [14]. Other merits in terms of chemical properties include high flash point, high cetane number, low viscosity, high lubricity, low emissions of carbon monoxide and sulfur dioxide.

Biodiesel today is prominently produced by transesterification of triglycerides of either refined edible or non‐edible oil using either methanol, ethanol, butanol or amyl alcohol [15] coupled with a catalyst such as alkali, acid or enzymatic based. Methanol is preferred due to cost, short chain alcohol, reacts quickly and is easily dissolved into the reaction medium [15]. This reaction takes place in temperatures of 60℃ to 80℃, with the neutralization of the glycerol and the FAME by a catalyst and separated before purification. The reaction for conversion of fatty acids during transesterification is catalyzed by acids, bases, biomass and bio‐waste catalysts or enzymatic catalysts [11, 16]. The enzymatic catalysts are the most effective for biodiesel production; however, their reaction time is slow while the cost is prohibitively high [1, 6, 17].

For industrial scale production homogeneous base catalysts are most commonly chosen for biodiesel production. Examples include NaOH, KOH and phosphoric acid, these catalysts offer many advantages such as high catalytic activity [18], shorter reaction time, modest operating conditions, low cost, and availability [19]. Nevertheless, homogeneous base catalysts have a reaction that is highly sensitive to free fatty acids and water. Therefore, during reaction, they form soap and large volumes of wastewater hence increased costs of production and operations. These qualities also make these catalysts environmentally unfriendly [19].

Homogeneous acid catalyst such as sulfuric acid (H_2_SO_4_), HCL, and phosphoric acid (H_3_PO_4_) perform better than homogenous base catalysts in high FFA feedstocks such as waste cooking oil, animal fats and most of the crude vegetable oils. Other merits include lack of sensitivity to water and FFA and an ability to double action in transesterification and esterification processes without forming soap by products [19, 20]. However, low reactivity (4000 times slower) is a major obstacle to commercialization. Additionally, homogeneous acid catalysts are corrosive and highly acidic in nature.

In general the cost of production of biodiesel and biofuel products is very high as the cost of raw material and processing are key determinants which affect cost and pricing [21]. In other words, development of novel approaches is critical to reducing processing and material cost. Among the novel solutions proposed is the use of heterogeneous catalysts, which offer an alternative solution to previous catalysts. Large‐scale commercialization has not been widespread in the energy sector, but since 2006 a pilot commercialized 160, 000 tonne/year processing plant operates in France. Catalysts are important in transesterification reactions due to the advantage of reducing reaction time and ease of product separation as they do not take part in the reaction neither are they consumed during processing.

Currently in the market there are a number of catalysts such as metal oxides, mixed oxides and hydrotalcites [22], while for acidic operation they include transitional metal oxides, ion exchange resin, carbon based catalysts and zeolites. Among the demerits of this type of heterogeneous catalysts is its three phase reaction system leading to diffusion which inhibits reaction [1]. Additionally this three phase system inhibits mass transfer efficiency which is kinematically important for reaction rate [23]. Other demerits include low number of active sites, micro porosity, leaching, toxic, expensive, non‐renewable and environmentally unfriendly [24].

Biomass based heterogeneous catalysts have gained considerable attention due to high availability and reactivity for biodiesel production. These have been reported in a number of studies including cocoa pod husk [25]; palm kernel shells [26]; Musa paradisiacal peels [27], Musa gross Michel peels [28], ripe plantain peels [29], *Musa balbisiana* colla peels and underground stem [30, 31], tucuma peels [32], banana peels and cocoa pod husk [33], wood ash [34], *Lemna perpusilla* torrey [35], rubber seed shell [36], camphor tree [37], gasifier bottom ash [38], and rice husks [19].

## 2 CATALYST CLASSIFICATION

A catalyst is a substance that changes the chemical kinetics of a reaction but never changes its thermodynamic in the reaction [39]. Catalysts in a transesterification reaction increase the rate of reaction hence increase the yield of the final product. The catalysts applied in transesterification process are classified into four main groups i) homogeneous, ii) heterogeneous, iii) enzymatic catalysts (biocatalysts) and iv) non‐catalytic processes which operate with supercritical conditions.

### 2.1 Homogeneous acid catalyst

These are the most commonly and preferred catalysts in biodiesel production due to their cost, availability, and faster speed of reaction. Homogeneous catalysts include both acids and base types. Examples include H_2_SO_4_, NaOH and KOH. Various studies conducted have shown that catalysts employ many mechanisms in their reaction but their main mechanism lies in the nucleophilic attack on the carbonyl group [40]. Nevertheless homogeneous catalysts when applied present a number of demerits such as increased cost, excessive use and high wastage of catalyst, generation of large volumes of wastewater, difficulty in separating the final product, sensitivity to thermal changes which cause them disintegrate below 150°C [41]. Table 1 shows biodiesel production using different types of solid acid catalysts, feedstock sources tested, preparation methods, reaction operating conditions and references. The main highlights of the table is the low temperature of reaction, the relationship between catalyst weight and FAME productivity as functions of the normalized time yields and comparative high‐normalized time yields as shown in the table.

**TABLE 1:** Biodiesel production over solidacid catalysts

Catalyst	Feedstocks	CPM	Reaction operating conditions						Ref
			TR	RT	CW	FP	MR	NTY	
Cs_2.5_H_0.5_PW_12_O_40_	Sesame oil	Precipitation	260	1	3	0.30	40:1	90	[42]
CsHPW	Oleic acid‐ Soybean mixture	Precipitation	200	10	3	0.30	20:1	90	[43]
Mn_3.5_xZr_0.5_yAlxO_3_	WCO	Co‐precipitation	150	5	2.5	0.30	14:1	75	[44]
Anion/cation exchanged resin	Pure triolein	Neutralization	50	4	4	0.24	10:1	96	[45]
SiO_2_^‐^SO_3_H/COFe_2_O_4_	Rambutan oil	Co‐precipitation	65	5	5	0.19	20:1	95	[46]
HPW‐PGMA‐MNPs	Greases	Direct mixing	122	24	4	0.24	33:1	96	[47]
SiO_2_^‐^Pr‐SO_3_H	Acid oil	Direct mixing	100	8	4	0.24	15:1	96	[48]
SO^2‐^_4_/TiO_2_‐SiO_2_	Palm fatty acid distillate (PFAD)	Impregnation	150	3.12	2.97	0.31	5.85:1	92.07	[49]
MPD‐SO_3_H‐IL	Jatropha oil	Co‐polymerization	160	8	6	0.15	50:1	90	[50]
AlCl_36_H_2_O	Brown grease	Hydrothermal	42	<4	2	0.43	10:1	86	[51]
OMR‐[C_4_HMTA][SO_3_H]	Brown grease	Co‐polymerization	65	5	0.05	19	40:1	95	[52]
Carbon derived catalyst	*Calophyllum inophyllum* oil	Pyrolysis	180	5	7.5	0.12	30:1	90	[53]

CPM, Catalyst preparation method; CW, catalyst weight measured in grams and expressed as (NTY/FP); FP, FAME productivity (g)/t (hours) expressed as normalized time yield divided by the catalyzed weight (NTY/CW); MR, molar to oil ratio; NTY, Normalized time yield is an expression of CW/FP as a percentage (CW/FP*100); Ref, References; RT, reaction time (hours); TR, temperature of reaction (℃).

Application of alkalized homogeneous catalysts in transesterification provide a 4 000 times faster reaction than acid based catalysts [54]. Among the commonly used alkaline catalysts in the production of biodiesel are NaOH, KOH and sodium methoxide. Other alkaline catalysts utilized in the production of biodiesel include sodium ethoxide, potassium methoxide, sodium peroxide and carbonates. According to yield of biodiesel production, using alkaline catalyst CH_3_ONa or CH_3_OK are more effective and suitable as they easily dissociate into CH_3_O^–^ and Na^+^, and K^+^, respectively.

### 2.2 Heterogeneous solid base catalyst

Solid base catalysts offer a high specific surface area with a high concentration of basic sites, which ensures high catalytic activity. The commonly used solid base catalysts in terms of activity and strong base sites include single metal, mixed metal, doped metal oxides, alkali earth metals and transitional metal oxides. The base sites of most of the solid base metal oxides catalysts come from metal ions containing Lewis acid and Bronsted base sites. Single base metal oxide include MgO, CaO, SrO, and BaO [55]. Solid base catalysts give the same yield at low temperatures and within a short time [56].

However, the major limitation of solid base catalysts regarding high FFA (≥2%) is that they form soaps. CaO catalysts are commonly used in the production of biodiesel due to their low cost and high reactivity [57]. However, other single metal oxides such as MgO as base catalysts are weak and are less soluble in alcohol, although with an increased calcination temperature to 873 k their strength is increased [58]. Experimental work shows that CaO as a catalyst is more active compared to MgO due to its strong basic sites. On the other hand, alkaline earth metals oxides are effective catalysts for the production of biodiesel in low fatty acids under mild operating temperatures [59].

A number of studies suggest that to increase stability and catalytic activity doped metal oxides should be used [40, 60–62]. Doping metal oxides improves surface area, strength of the catalyst and pore size of the material synthesized. For example doped lithium was effective with a 23% w/w of CaO the yield almost equaling 100% within a reaction time of 20 min [63].

### 2.3 Heterogeneous solid acid‐base catalyst

Heterogeneous solid catalysts are considered effective due to the presence of Lewis acidity [35]. Heterogeneous catalysts besides being recyclable operate with little deactivation during transesterification [3, 34, 35]. Heterogeneous catalysts include sulphated zirconia and tungstated zirconia [98], zeolites and zeotype materials, mixed oxides (silica alumina), sulfonated polystyrene, ion exchange resins and hetero‐polyacids [65, 66]. Zeolites and zeotype are naturally occurring in the form of crystalline aluminosilicates, interlinked with oxygen atoms; thus, forming a three dimensional molecular pore structure of equal sizes [67].

Zeolite pores have the ability to absorb molecules their own size but excluding larger molecules, reacting like sieves. This structure helps zeolites to exchange ions through production of negative ions with catalyst structures to improve catalytic activity. Another feature of zeolites is the ability to improve adsorption due to the strong electric field that is developed by cations within the pore active sites of the catalyst [68].

Due to development in research of recyclable solid acid catalysts, the production cost of biodiesel has reduced making it a favorable alternative to oil. Heterogeneous solid catalysts contain both Bronsted type (sulfonic acids) and Lewis type (mixed sulfated oxides) [66] giving them a combined advantage over base catalysts and mineral acids. In other words, heterogeneous solid acids can be used to conduct both transesterification and esterification simultaneously. Heterogeneous solid catalysts are also insensitive to moisture and high FFAs, which is peculiar to low grade feedstocks. This allows utilization of cheaper feedstocks which cuts out acid pre‐treatment production costs [60]. The elimination of this step in production of biodiesel makes it possible to minimize corrosion among acid feedstocks species. Other advantages of heterogeneous solid acid catalysts include selectivity, recyclability and regeneration lower deactivation contamination and easy separation of products [69, 70].

Studies indicate that zeolite transesterified reactions form undesirable by products such as di‐methyl ether due to dehydration of the methanol [71]. Their performances rely heavily on the interchange between their acid strength and their hydrophobicity because of their high SiO_2_/Al_2_O_3_ ratio. In other words, lower ratios lead to a high acid strength and vice versa. Nevertheless poor performance has been reported in some literature reviewed for some high aluminum content zeolites [72].

### 2.4 Biocatalyst and bio‐waste heterogeneous catalysts (green’ catalysts)

Due to the increase in the global demand of biodiesel and biofuels estimated at 5% annual growth [73], the production of biodiesel and biofuels, using biocatalysts, has become increasingly critical. Waste derived from industrial end processes can aid in the development of low cost and eco‐friendly heterogeneous solid base catalysts [74, 75]. For example most calcium enriched sources such as egg shells, mussel shells, cockle shells, oyster shells, fish scales, animal bones, and ash derived from plant waste are feasible sources of catalysts [76]. In the literature reviewed, many researchers have reported the critical importance of calcium obtained from waste material and converted into calcium oxide, which is a versatile heterogeneous catalyst [77, 78].

In the literature surveyed most enzyme catalyst are reported sensitive to water which makes it hard to apply polar substance such as water, methanol, glycerol and phospholipids [10]. These substances inhibit enzymatic catalytic activity during the process of transesterification. The researchers reviewed have mitigated this problem by use of alcohol and other organic substances by step‐wise addition of methanol. Table 2 is showing a summary of biocatalyst enzymes used in the transesterification of oil obtained from non‐edible feedstocks the reaction operating conditions and their references.

**TABLE 2:** Transesterification of non‐edible oils using biocatalyst enzymes

Catalyst	Feedstock	Reaction operating conditions								Refs
		ST	MR	TR	RT	WC	CW	FP	NTY	
*Burkholderia cepacia*	*Jatropha curcas*	Ethanol	10:1	35	24	4.49	1	1	100	[79]
*Candida parapsilosis* lipase	*Jatropha curcas*	Methanol	2:1	30	8	10	0.25	3.22	80.50	[80]
	*Calophyllum inophyllum*	Methanol	12:1	35	25	15	20	0.04	92	[81]
	*Pistacia chinensis* bge seed oil	Methanol	5:1	37	60	20	2.5	0.36	90	[82]
		Methanol	5:1	37	60	20	7	0.13	91	[82]
Lipozyme TL IM	Castor oil	Methanol	3:1	45	24	–	15	0.04	60	[83]

CW, catalyst weight measured in grams and expressed as (NTY/FP); FP, FAME productivity (g)/t (hours) expressed as (NTY/CW); MR, molar to oil ratio; NTY, normalized time yield is an expression of CW/FP as a percentage (CW/FP*100); Ref, references; RT, reaction time (hours); ST, solvent type used; TR, temperature of reaction (℃); WC, water content (%) by volume.

Biocatalysts have been described as the perfect replacement for chemical catalysts performance and reactions [84]. As natural occurring lipases, biocatalysts have the ability to perform both esterification and transesterification processes for biodiesel production. Biocatalysts are obtained from enzymes which are living organisms but which promote chemical reactions without being affected themselves [85, 86]. In the production of biodiesel through transesterification reaction two types of biocatalysts are employed, extra‐cellular and intra‐cellular lipases [87]. The former refers to enzymes obtained from microorganism's broth and purified for use as catalysts, while the latter lipases remain either in the cell producing walls or inside the cell itself. Biocatalysts have a number of advantages over other catalysts such as simplified production process, lower energy consumption, higher product purity of glycerol, less soap formation during reaction, easy catalyst separation, and reuse of immobilized enzymes [6].

**FIGURE 1:**
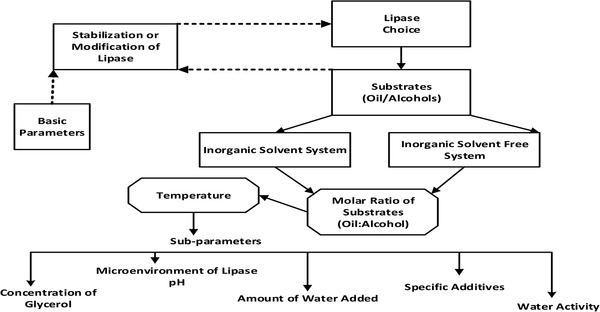
Factors affecting biodiesel yield using enzymatic catalysts [96]

On the other hand, the disadvantages include lower reaction rates, higher costs of lipase and production, and loss of catalytic activity caused by enzyme inhibition [88]. Biocatalysts are identified from a number of bacteria species such as *Pseudmonas flourescens, Pseudomonas cepacia, Rhizomucor miehei, Rhizopus oryzae, Candida rugosa, Thermomycis linuginosus* and *Candida antarctica*. Among the highly studied immobilized lipases are Novozym 435, Lipozyme TL IM, Lipozyme RM IM, and Ps‐C [89, 90, 91, 92]. Figure 1 shows factors affecting enzymatic catalysts during transesterification. This is a summary of factors, which influence and affect enzymatic catalysts during transesterification. Figure 1 breaks down these factors into two main blocks of parameters of basic parameters and the second block is sub‐parameters. The figure is primarily meant to show the relationship between these factors and how they interaction during reaction of enzymes in transesterification.

Biocatalysts in the production of biodiesel present additional demerits such as the use of free enzymes, which are not stable under many reaction conditions of transesterification. It also presents a very difficult situation during separation of the reaction mixture hence making recycling of enzymes a near impossibility [93]. In order to overcome these limitations researchers have identified enzyme immobilization onto supports in solid carriers. These carriers increase chemical and thermal stability while protecting enzymatic molecules from denaturation [94]. However, it is important to also note here that there is a possibility of enzyme shape change with the support matrix, detachment and a drop in catalytic activity coupled to the support material cost [95].

For the improved production of biodiesel, a number of methods have been designed to help in lipase immobilization. Immobilization of lipase current research uses a number of techniques, which have been developed overtime. Enzymes, as biocatalysts, are prepared via immobilization with inert materials, allowing resistance to change in pH, moisture level and temperature. These methods include adsorption, covalent bonding, entrapment, and encapsulation and cross‐linking [97]. These methods will not be discussed at length but the reader can refer to the references provided here for further study. The main aim is to improve lipase stability for the process of biodiesel [98, 99]. Second, biocatalysts help to overcome the difficulties associated with the use of heterogeneous catalysts. These complications include high‐energy requirements, high cost of production and complicated procedures of use [10].

### 2.5 Nanocatalysts as heterogeneous catalysts

Nanocatalysts have gained significant prominence regarding biodiesel production due to their high efficiency compared to conventional catalysts. Nanoparticles for both inorganic and organic support for enzymes immobilization fall into two groups; the magnetic and non‐magnetic. The non‐magnetic group support covers silica, polystyrene, chitosan and polylactic acid divided into two groups’ synthetic polymers (chemical polymers) and biopolymers, obtained from renewable materials [100]. Silica is the leading support material for enzyme immobilization support. It is preferred due to its high thermal and chemical resistance, excellent mechanical properties; it is cheap, non‐toxic and offers good compatibility. Additionally silica provides good adsorption properties as it has a high surface area and porosity hence offers minimum diffusion [101]. Inorganic nanoparticles include nano‐metals such as all metal oxides, zirconia, Titania, alumina, gold and silver. These Nano‐metals have shown high stability, good mechanical resistance and good adsorption capabilities. The nano‐metals of this groups show support for different groups and classes of enzymes in various reaction conditions, as they remain inert [100, 102].

Although the non‐magnetic nano‐particles that support enzymes disperse well in the reaction solution. This nevertheless creates a difficulty in reuse of biocatalysts as separation from the reaction mixture high speed centrifugation hinders it [103]. Research in much literature reviewed here show that to overcome recovery issues related to nanoparticles can be achieved by attaching magnetic oxide (magnetite Fe_3_O_4_) to enzyme molecules [104, 105]. Magnetite iron oxide offers low toxicity, biocompatibility, large surface area and an abundance of the hydroxyl group on the surface, hence functionalization of a strong covalent bonding of the enzymes. Other important nanoparticles according to literature surveyed include carbon Nanotubes [106, 107, 108, 109], Nanofibers [110], and Nanocomposites [111, 112].

Nanocatalysts boast a high surface area, increased catalytic activity, high stability, superior resistance to saponification, efficient surface to volume ratios and high reusability [113]. This reduces diffusion limitations while enabling enzyme loading. Sodium titanate nanotubes have been studied in relation to the production of biodiesel. Sodium titanate was found to have 200 M^2^/g and a pore volume of 0.61 cm^3^/g the yield obtained was 98% with a catalyst loading of 1% and a methanol molar to oil ratio of 20:1 [114]. A number of hybrid studies have been undertaken with nanocatalysts using lipases, for example [115, 116, 117, 118].

Nanocatalysts can be processed using various systems such as self‐propagating high temperature synthesis, microwave combustion, conventional hydrothermal, microwave hydrothermal, microwave solvothermal, sol‐gel technique, co‐precipitation, impregnation, gas condensation, chemical vapor deposition, electrochemical deposition, vacuum deposition and evaporation [119, 120]. However, for this techniques to be effectively applied it is critical that characterization of the catalysts is undertaken before application in biodiesel production. Table 3 shows different nanocatalysts used for biodiesel production from different feedstock sources and their reaction operating conditions.

**TABLE 3:** Different nanocatalysts used for biodiesel production from different feedstock sources

Catalyst	Feedstock	Reaction operating conditions							Ref
		ST	MR	CW	TR	RT	NC	NTY	
NaAlO_2_/γ‐AlO_3_	Palm oil	Methanol	20.79:1	10.89	64.72	3	1‐6	97.65‐93.29	[5]
25%MoO_3_/B‐ZSM‐5	Oleic acid	Methanol	20:1	3	160	6	1‐6	98‐93	[122]
CaO/CuFe_2_O_4_	Chicken fat	Methanol	15:1	3	70	4	1	94.52	[123]
KOH/Fe_3_O_4_@Al_2_O_3_	Canola oil	Methanol	12:1	4	110	4	1‐6	98.80‐88.40	[124]
Mg/MgFe_2_O_4_	Sunflower oil	Methanol	12:1	4	110	4	1‐6	91.20‐82.40	[125]
Cr/Ca/ℽ‐Al_2_O_3_	Cooking oil	Methanol	18:1	6	65	3	1‐6	92.79‐78.29	[126]
MgO/MgAl_2_O_3_ (untreated and treated with plasma	Sunflower oil	Methanol	12:1	3	110	3	1	95.70‐96.50	[113]
MgO/MgAl_2_O_3_ (untreated and treated with plasma	Sunflower oil	Methanol	12:1	3	110	3	5	79.30‐91.10	[113]
ℽ‐Al_2_O_3_/KI	Palm oil	Methanol	14:1	4	60	4	1‐11	98‐79	[126]
Ca/ℽ/Al_2_O_3_	Corn oil	Methanol	12:1	6	65	5	1‐5	87.89‐34.64	[127]
Cs/Al/Fe_2_O_4_	Sunflower oil	Methanol	14:1	6	58	2	1‐4	95‐88	[128]

CW, catalyst weight measured in grams and expressed as (NTY/FP); MR, molar to oil ratio; NC, number of cycles the catalyst is used; NTY, normalized time yield is an expression of CW/FP as a percentage (CW/FP*100); Ref, references; RT, reaction time measured in (hours); ST, solvent type used; TR, temperature of reaction (℃).

Among the commonly used characterization methods for nanocatalysts in the literature surveyed is x‐ray diffraction (XRD), which allows catalyst composition and crystallinity characterization. This is allowed by scanning electro‐microscopy (SEM) to prepare the nanocatalysts morphology for determination. Another common method employed is Fourier transform infrared (FTIR) spectroscopy, which helps in determining assimilation phases for specific surface area calculation and thermogravimetric analysis (TGA) during the decomposition of catalyst samples [62, 121].

## 3 SOURCES OF BIOMASS HETEROGENEOUS CATALYSTS

Heterogeneous carbon‐based catalysts are derived from lignocellulose. They are non‐edible biomass, and thus renewable sources of biofuel, biochemical and industrial remediation applications [129, 130, 131]. Although they are cheap with exceptional physicochemical advantages, non‐corrosive and easy availability as natural resources [132, 133, 134, 135], efficient conversion of hydrocarbons (HCs) into commercially durable and viable materials is a challenge [136, 137, 138]. Table 4 is a summary of various types of catalysts from waste feedstocks and their derived catalysts used in transesterification. Table 4 shows also the widespread use of waste resources as feedstocks for catalysts and the type of catalysts derived. The table includes the reaction operating conditions and results obtained by different researchers using waste derived catalysts and their references.

**TABLE 4:** Summary of various types of waste catalysts in transesterification

Feedstock	Catalyst	Reaction operating conditions								Ref
		CT	TR	MR	RT	CW	FP	NTY	NC	
Mollusc shells										
Mudcrab (Scyllaserrata)	CaO	900	65	0.5:1	2.5	5	0.19	95	15	[142]
Biont (turtle)	KF‐CaO	500	70	9:1	3	3	0.32	96	–	[143]
Shrimp	KF‐CaO	450	65	9:1	3	2.5	0.35	87.50	–	[144]
Oyster	CaO	700	65	6:1	5	25	0.03	75	–	[145]
Snail	CaO	900	60		8	2	0.49	98	–	[146, 147]
Chicken	CaO	1000	65	9:1	3	3	0.31	93	13	[148, 149]
Chicken	CaO	900	60	9:1	3	3	0.32	96	14	[150]
Ashes										
EFBA	KOH	550	65	15:1	20	1.5	0.65	97.50	5	[151]
KOH/EFBA	KOH	550	65	15:1	15	0.75	1.32	99	5	[151]
Coal fly ash loaded with KNO_3_	K_2_O	500	160	15:1	5	15	0.05	75	3	[152]
Coal fly ash loaded KNO_3_	K_2_O	500	70	15:1	8	15	0.05	75	–	[153]
Coal fly ash loaded egg shell	CaO–Al_2_O_3_ and SiO_2_	1000	70	6.9:1	5	1	0.96	96	16	[154, 155]
Rocks										
Alum	KAl(SO_4_)_2_	550	170	18:1	12	7.09	0.13	92.17	–	[156, 157]
Dolomite	CaMg(CO_3_)_2_	850	67.5	6:1	3	3	0.30	90	5	[158]
Dolomite	CaMg(CO_3_)_2_	800	60	30:1	3	6	0.16	96	7	[159]
Calcite	CaCO_3_	800	60	30:1	3	6	0.07	42	–	[159]
Bones Rohu fish (*Labeo rohita*) bone	β‐Ca_3_(PO_4_)_2_	997.42	70	6.27:1	5	1.01	0.96	96.96	6	[160]
Sheep bone	Hydroxyapatite	800	65	18:1	4	20	0.04	80	5	[24]
Cuttlebone	CaCO_3_	800	60	30:1	3	6	0.04	24	–	[161]

CT, calcination temperature in (℃); CW, catalyst weight measured in grams and expressed as (NTY/FP); FP, FAME productivity (g)/t (hours) expressed as (NTY/CW); MR, molar to oil ratio; NC, number of cycles the catalyst is used; NTY, normalized time yield is an expression of CW/FP as a percentage (CW/FP*100); Ref, references; RT, reaction time in (hours); TR, temperature of reaction in (℃).

As catalysts their catalytic activity can be enhanced by incorporating the oxygenated capabilities of the carbonyl group (C=O), sulfonic acid (SO_3_H) hydroxyl (OH), and the carboxylic group (COOH) [132, 139]. Addition of COOH to HC catalysts enhances the hydrolysis process of biomass under low temperature. The COOH group has greater water solubility characteristics and active sites, which increase adsorption of biomass via the hydrogen bond [140, 141]. For example in the hydrolysis of β‐1,4‐glucans to glucose on air iodized carbon, catalysis reaches a 70% yield in the presence H_3_PO_4_ at 230℃.

### 3.1 Waste shell

Waste shells contain calcium carbonate and a high potential to produce heterogeneous catalysts for the synthesis of biodiesel. For example, mollusc shells were being researched as early as the late 17th century [162] are found on many shorelines and have remarkable physical properties of 95 to 99% crystalline calcite or calcium carbonate, 33% protein and 66% hydroxyapatite [163]. As heterogeneous catalyst sources, mollusc shells have been investigated by a number of researchers, for example [164]. These authors investigated the use of crab shells (*Scylla serata*) as synthesis catalysts in palm olein oil and in this study they reported that crab shells contain CaO [164, 165]. When the shells were calcined at 900℃ for 2 h they transesterified chicken fat at a yield of 98.5 to 99% in a reaction time of 3 h.

Another investigation using waste shells involved Biont shells (turtle shells) and was conducted by [141, 166]. The authors prepared the shells at 500℃ via different treatments such as impregnation in KF for 6 h and catalyst load of 25% wt. % and thermal activation of 300℃. The study reported that strong basicity formed after impregnation with KF; thus, concluding that Biont shells from waste provided excellent heterogeneous catalyst with activity and stability under mild reaction operating conditions. A similar finding has been reported using Biont waste shells but with changed operating conditions, with a calcination temperature of 450℃, thermal activation of 250℃ reaction time of 3 h and catalyst load of 25 wt. % compared to the previous experiment. This experiment reported a conversion yield of 89.1% [167]. Many researchers have undertaken waste shell work [78, 165, 168, 169, 170, 171, 172, 173, 174].

### 3.2 Biomass ashes (fly ash, empty palm fruit bunch‐based boiler ash)

Organic compounds contain high quantities of carbon, oxygen, metal salts such as potassium, sodium, magnesium and calcium [175]. During combustion these compounds separate while reducing the C and O content leaving the alkali metal oxides such as CaO, K_2_O, and MgO as active ingredients in the ash [30]. As a result waste plant and tree sources as catalysts have drawn a lot of attention in the past decade, attracting many researchers in biodiesel synthesis due to the catalytic nature of these compounds [176, 177]. Table 5 is summary of elemental material composition found in different ash feedstocks by their weight percentage and references. These different ash feedstocks have been discussed in extensively in Section 3.2 and this culminates into Table 5 as the elemental composition of different ash feedstocks.

**TABLE 5:** Elemental composition of different ash feedstocks by weight

Ash feedstock	Elemental compositions of ashes (wt. %)										Ref
	K	Si	Ca	O	C	Mg	P	Al	Cl	Na	
Oil palm ash	40.59	2.63	–	29.36	14.56	–	0.76	0.73	0.50	7.07	[178]
Wood ash	5.7	21.5	17.8	–	–	4.5	0.50	1.2	–	5.7	[179]
*L. perpusilla* Torrey ash	11.32	82.52	5.10	–	–	–	–	–	1.10	0.53	[177]
Cocoa pod husk ash	42.9	3.25	–	–	4.44	1.21	–	–	43.57	2.92	[30]
Husk rice ash	0.44	25.77	6.84	66.95	–	–	–	–	–	–	[180]

The use of biomass ashes as catalysts in the transesterification process produces biodiesel with a yield of 96% at room temperature for 3 h with the biodiesel quality free of sulfur and with a high cetane number [30, 181]. Besides plant and tree biomass ash, fly ash is also an inorganic waste that can be classified as an ash resource. The chemical composition of fly ash is around 55% SiO_2_, 30% Al_2_O_3_ and other oxides [182]. In the literature reviewed, a number of researchers have utilized fly ash as support for loading CaO during chemical production reactions [36] with great success. In the literature surveyed, this support gave higher FAME conversion and catalyst reuse of three cycles without loss to catalytic activity. In other words, the high amount of SiO_2_ and Al_2_O_3_ is a potential of low cost catalyst and in the last decade fly ash supported CaO has been utilized as a recyclable solid base catalyst by researchers such as [183, 184].

### 3.3 Rocks

This includes dolomites lime, clays etc. For example, dolomite rock is a natural rock comprising of alternating layers of calcium carbonate and magnesium carbonate. Through calcination at 750℃ the calcium carbonate and magnesium carbonate decompose forming CaO and MgO, respectively, [167]. Both dolomite (CaMg (CO_3_)_2_) and calcite (CaCO_3_) can be transformed into CaO and MgO using thermal processes for decomposition.

In the last decade, Dolomite has been utilized to produce biodiesel by transesterification of the C_4_C_8_ olive oils. At present, the lower cost of dolomite makes it useful in the cement industry and in landfilling. The catalyst obtained from dolomite shows increased surface area compared to the uncalcined natural dolomite rock and a number of literature reviews show increased studies for calcined dolomite rock such as [185, 186, 187]. The maximum yield obtained so far using dolomite in literature‐reviewed ranges from 92 to 99% FAME conversion. Enhancement of the dolomite rock using liquid catalysts with a variety of modification using hydration, and with dehydration to change the morphology of dolomite different, have been reported [158, 188] with a FAME yield of 93 to 97.4% conversion.

### 3.4 Bones

Animal waste bones have been reported to contain low cost heterogeneous catalyst material for the production of biodiesel. Waste animal bones are products of the meat industry and comprise hydroxyapatite (Ca_10_ (PO_4_)_6_ (OH)_2_, HAP) as the main element. In the literature reviewed, the use of animal waste bones as sources of catalysts has been reported widely in the last decade. For example, the use of fish bones for biodiesel synthesis [189, 190, 191, 192]. In their findings, these authors found high catalytic performance of up 96% conversion of FAME using calcined waste fish bones, due to the formation of β‐tri‐calcium phosphate.

Other animal waste bones, which have been utilized for the synthesis of biodiesel in the literature surveyed, include sheep bone, which gave a conversion rate of 96.78% at 800℃ calcination temperatures, bovine bone waste [24, 193], chicken waste bones [22, 194] and cow waste bones [195, 196]. Besides utilization as catalysts, studies indicate that animal waste bones can also double as support for other catalysts. This is due to their porous natural structure and adsorption capabilities [197]. Many heterogeneous catalysts have been supported using hydroxyapatite in research such as in [179, 197, 198]. Besides supporting copper‐based catalysts animal waste bone catalysts can support potassium salts such as K_2_CO_3_ and KOH for the synthesis of other catalysts [198, 199, 200]. Although the results in terms of yield is satisfactory at 59.90% conversion rate, nevertheless for the unreacted triglycerides using calcined fish scales obtains a yield of 98.4%. For potassium support, the experiment shows high catalytic activity above 96% due to high basicity [167].

### 3.5 Biochar derived heterogeneous catalysts

As a biomass resource, biochar production comes from a variety of sources such as agricultural residues, and municipal solid waste (MSW) (using fast/slow pyrolysis, gasification and hydrothermal carbonization) [201, 202, 203, 204]. The interest in biochar is the number of chemical reactions, occurring during the formation of biochar. These reactions include decomposition and depolymerization of polymeric compounds, rearrangement, dehydration, decarboxylation, intramolecular condensation and aromatization [205]. Biochar, after undergoing these chemical reactions, can be activated via physical or gas activation, chemical treatment or impregnation to modify its functionality and chemical properties [206, 207].

Among the activation methods of biochar, the chemical technique holds sway owing to the high yield, lower temperatures of reaction, reduced activation time and, importantly, high mesoporous formation [208]. The commonly used chemicals, which induce pores in biochar, include NaOH, KOH, phosphoric acid (H_3_PO_4_), ferric chloride (FeCl_3_) [209]. As a result, biochar has gained attention due to its many merits such as low cost, availability, high adsorption, stability and high specific surface area [204]. Besides, biochar also improves air and environment quality caused by open field burning during agricultural land preparation for planting [210, 211]. A number of researchers have utilized biochar in the synthesis of biodiesel such as hardwood biochar as a catalyst using concentrated sulphuric acid (H_2_SO_4_). In this study the authors report that Sulfonation improves the textural properties of any biochar catalysts with results indicating an 89% and 92% conversion rate for non‐sulfonated biochar and vice versa, respectively, for canola oil at 120℃ for 24 and 15 h. Other studies, which have yielded interesting reports and high yields from biochar‐based biomass materials, include studies by [212, 213, 214, 215].

## 4 CATALYST PREPARATION METHODS

Heterogeneous catalyzed processes during transesterification are very complex in nature as they occur in three phases. These phases include a solid phase (heterogeneous catalyst phase) and two immiscible liquid phases (oil and methanol). Additionally there are side reactions such as saponification of glycerol and methyl esters and neutralization of FFAs [26, 216, 217].

In the literature surveyed, a number of methods have been suggested for the preparation of solid catalysts. The methods described include hydrothermal synthesis, impregnation, thermal treatment, and physical mixing. The choice of method used to prepare catalysts depends on the physical and chemical properties of the final product [218].

### 4.1 Selection of catalysts

Structurally, metal oxides comprise positive metal ions called (cations) with Lewis acidity which makes them electron acceptors and negative ions (anions) which are proton acceptors (Brønsted bases). These characteristics allow metal oxides to have a great adsorption influence in methanolysis of any oil undergoing transesterification for biodiesel production. For example, it provides sufficient adsorptive sites for methanol in which the O‐H bond breaks forming methoxide anions and hydrogen cations.

The literature reveals a variety of catalyst preparation methods. For example, certain quantities of water improve catalytic activity of CaO in the production of biodiesel [219]. In other words, O^–2^ in the presence of water on the surface of the catalyst extracts H^+^ to form OH^–^ with the OH^–^ subsequently extracting the H^+^ of the methanol to form methoxide anions, which are the catalyst of the transesterification process [220]. Another notable report by researchers is by [64]. These authors observed that the adsorption of methanol was a key reaction rate determined in catalyzed reactions such as MgO and La_2_O_3_, while the surface reaction step for catalysts with higher basicity was rate determining for BaO, CaO, or SrO catalysts.

Other studies have investigated over 13 different metal oxides containing oxides such as calcium, barium, magnesium and lanthanum for producing biodiesel through transesterification. The results show that calcium based catalysts show a higher catalytic activity during the process of transesterification. Calcium oxide is frequently used for biodiesel production synthesis due to its availability and low cost of purchase and minimal toxicity compared to other catalysts [64, 221].

### 4.2 The calcination method

The most common method of preparation of catalysts derived from biomass is calcination. Calcination involves use of thermal treatment in the absence of air and oxygen to break biomass into smaller components [99]. Calcination can be applied in a wide range of temperatures ranging from 300°C to 1000°C based on the type of biomass feedstock. During thermal treatment and upon the combustion CaCO_3_ breaks down to CaO while releasing CO_2_ gas. Table 6 is a summary of biomass derived solid base catalysts using the calcination method for biodiesel production through transesterification. The table provides a summary of types of biomass calcined for catalyst production and the type of feedstock. The table also gives the reaction operating condition in making this biomass derived catalysts and their references.

**TABLE 6:** Biomass‐derived solid base catalyst by calcination method for biodiesel production

Type of biomass	Type of feedstock	Reaction operating conditions							Ref
		CT	RT	TR	CW	FP	MR	NTY	
Waste shell									
*Labeo rohita*	Soybean oil	600–1000	2	70	1.01	0.4890	6.27:1	97.70	[177]
Oyster shell	Soybean oil	1000	4	50	1	0.2130	10:1	85	[222]
Golden apple snail	Palm olein oil	800	2‐4	60	10	0.0930	18:1	93	[223]
*Meretrix venus*	Palm olein oil	800	2‐4	60	10	0.0920	18:1	92	[223]
*Amusium cristatum*	Palm oil	900	2	60	3	0.3100	8:1	93.00	[167]
Scallop waste shell	Palm oil	1000	4	65	10	0.0950	9:1	95.40	[146]
Crab shell	Sunflower oil	900	2	60	3	0.2770	6:1	83.10	[153]
Waste coral									
Coral fragment	Vegetable oil	700	0.5‐1.5	65	50	0.0196	15:1	98	[224]
Waste eggshell									
Eggshell	Palm olein oil	800	2‐4	60	10	0.0941	18:1	94.10	[223]
Duck eggshell	Palm oil	900	4	60	20	0.0465	9:1	92.90	[146]
Chicken eggshell	Palm oil	900	4	60	20	0.0472	9:1	94.40	[146]
Eggshell	Sunflower oil	900	2	60	3	0.3260	9:1	97.80	[153]
Animal bones									
Bovine bone waste	Soybean oil	350–1000	6	65	8	0.1212	6:1	96.96	[24]
Biomass ashes									
*Musa balbisiana* Colla ash T.	Peruvinia seed oil	–	0.5	32	20	0.0480	20:1	96	[169]
Tars and alkali ashes	Sunflower oil	600–800	12	80	10	0.0751	30:1	75.10	[173]
Coconut husk ash	Jatropha oil	250–500	1	45	7	0.1286	12:1	90	[30]
Activated carbon supported catalyst									
Fly ash/CaO‐derived eggshell	Soybean oil	1000	2	70	1	0.969	9:1	96.90	[152]
Cocoa pod husk ash/MgO	Soybean oil	650	4	40	7	0.141	6:1	98.70	[174]
Empty palm bunch ash	Waste cooking oil	30	3	60	17.3	0.0495	5:1	85.72	[225]

CT, calcination temperature in (℃); CW, catalyst weight measured in grams and expressed as (NTY/FP); FP, FAME productivity (g)/t (hours) expressed as (NTY/CW); MR, molar to oil ratio; NTY, Normalized time yield is an expression of CW/FP as a percentage (CW/FP*100); Ref, references; RT, reaction time in (hours); TR, temperature of reaction in (℃).

Calcination temperature plays a critical role in the development and formation of CaO catalyst surface morphology. Most waste material shells are non‐porous and therefore their developed size on the surface of the catalyst gives the total surface area of the prepared catalyst. In other words, catalytic activity of any prepared catalyst is dependent on the calcination temperature for its intensity in active sites [24]. The effect of calcination is extensively reported in a number of studies reviewed, for example [24, 99, 169, 224, 226, 227]. Many of these researchers report that at lower calcination temperatures of 600°C the surface showed a non‐uniform and aggregated arrangement due to amalgamation of elemental components such as Ca, Na, Mg, Si and Sc [169].

On the other hand, higher calcination temperatures of 700°C to 900 make CaO a major compound in the treated sample with different sizes and shapes of particles depending on the time of exposure. This means that a short holding time is a disadvantage as it may lead to underdevelopment of CaO; thus, affecting catalytic activity [224]. However, increased temperature, exceeding 950°C, reduces catalytic activity in the calcined sample due to the low pore volume and presence of micro‐pores, which curtail the quantity of active sites on the surface of the catalyst [99].

### 4.3 The hydrothermal method

Another commonly used method of catalyst preparation is called the hydrothermal method. This method uses a preheating order and solution mixing to bring about a difference in crystal morphologies. For example preheating Zn(NO_3_)_2_ solution forms ZnO nano‐rods with an average length and width of 230 and 38 mm. Hydrothermal synthesis preparation method is a reaction which dissolves and recovers materials that are insoluble under standard conditions by subjecting them to higher temperatures and pressure (HTHP) [228]. Among the key merits of a hydrothermal system are physical and chemical properties of high speed, high yield, low cost and eco‐friendly [229].

In a hydrothermal processing method, the value of the final product is close to the initial stoichiometric value. This is due to the higher surface contact and interaction between the solvent and the soluble particles. In other words increased reaction temperatures increases solubility of the particles due to reduced viscosity of the solvent. This also increases the mass transfer of the molecules hence increased particle movement hence rate of diffusion [229]. The hydrothermal catalyst preparation method produces highly crystalized nano‐particles with quite a narrow size distribution and high purity requiring no further calcination. In other words, pressure, time, the particle size, morphology and the crystal structure of a catalyst can be controlled [230].

A number of studies have been conducted using the hydrothermal method of catalyst preparation with success. Such as continuous hydrothermal synthesis using decanoil acid‐modified and oleic acid modified nano‐particles. Other researchers who have published in this area and reported their findings include [231, 232, 233, 234].

### 4.4 Impregnation method

The impregnation method (also known as capillary impregnation or dry impregnation) prepares the heterogeneous catalysts with the addition of a metal containing solution to a catalyst as support. Thereafter, the imbibed solvent is dried and removed from the substrate [218].

The impregnation preparation method is divided into two main methods, dry and wet. These two methods are distinguished by the manner in which the contacting of the solid occurs and the volume of the impregnation solution. In wet impregnation, an excess solution is used and separated after some time and the excess removed by drying. In incipient wetness impregnation (dry) the volume of the solution is equal or less compared to the pore volume of the supporting solid or the active solid phase [235, 236].

The literature reported a number of factors that affect the impregnation method such as temperature and the concentration of the initial solution [237, 238]. Temperature influences both the precursor solubility, solution viscosity and the wetting time [235, 236]. Impregnation preparation method has been used in a number of research using a KF/CaO nano‐catalyst by impregnation, which include [239, 240]. Some of these authors used impregnation to prepare a Li/ZnO catalyst by calcination for transesterification of soybean oil. In their experiment, the authors reported a correlation or a link between catalytic activity and catalyst properties. In addition, the authors reported that Li/ZnO as a catalyst provides good catalytic activity with its performance dependent on the load amount of lithium and the calcination temperature. These findings are corroborated in another study by.

The impregnation catalyst preparation method offers a number of merits such as being faster, less expensive and allowing control over the final product of the catalyst [241]. Nevertheless, it is one of the most difficult method of catalyst preparation as it is hard obtaining dispersion of catalyst components on the surface of the catalyst.

### 4.5 Sol‐gel method

The sol‐gel method can be described as similar to the precipitation method. The sol‐gel method is employed for catalytic feedstocks with materials that are colloidal in nature such as SiO_2_ [242]. Unlike conventional catalyst preparation methods the sol‐gel technique allows preparation of porous materials singularly while maintaining a homogeneous component distribution. It involves formation of a sol and a gel, which typically use colloidal dispersions or inorganic precursors. Nevertheless, the application of the sol‐gel preparation method is open to a wide variety of precursors, which take the form of an alkoxide (M(OR)n [243]. The sol‐gel preparation method, thus, uses two phases, the sol, which is a colloidal suspension made up of solid particles the gelation which is an interconnected gel of solid particles forming a continuous liquid throughout the phase [244, 245].

The main merits of using the sol‐gel preparation method include high yield, low operating temperature and low production costs [246]. In the literature surveyed, a number of researchers have used this method in reporting different studies such as [247, 248, 249, 250, 251, 252, 253, 254]. The sol‐gel method takes three main routes namely, hydrolysis, condensation and mixed condensation routes as show in the following Equations (1), (2) and (3).

(1) $$-MOR+H_{2}O\to -MOH+ROH,$$

(2) −MOH+ROM−→−MOM+ROH,

(3) $$-MOH+HOM-\to MOM+H_{2}O,$$

### 4.6 Co‐solvent preparation method

The co‐solvent method of catalyst preparation is one of the techniques used to enhance catalytic activity. A co‐solvent is applied to overcome the limitations of mass transport between methanol and the oil in order to speed up the catalyzed reactions [255]. In most co‐solvent catalysts, the preparation tetrahydrofuran is used as a co‐solvent for transesterification processes. In experimental work reported in the literature reviewed, a number of researchers have used this technique with success, including [145, 256–258]. In their findings, these authors report a high FAME yield of 90 to 98.5% conversion rate using the co‐solvent technique of catalyst preparation.

## 5 EFFECTS OF CHEMICAL AND PHYSICAL PROPERTIES OF HETEROGENEOUS CATALYSTS ON BIODIESEL PRODUCTION

Heterogeneous catalysts appear in solid form but act in the opposite way when in the liquid phase of homogeneous catalysts. Although in principle homogeneous catalysts are cheap, readily available, and offer high reaction rates and a shorter processing time in biodiesel production [3], their use of H_2_SO_4_ leads to high sulfur content that exceeds the specified quantity in the biodiesel standard of 10 ppm. On the other hand, NaOH and KOH when applied in the production and processing of biodiesel additional requires separation of product, recovery of liquid catalyst and product washing via acid neutralization. This last step increases the amount of waste water hence cost of production which is a major hindrance in commercialization of biofuels and biodiesel [10].

The introduction of heterogeneous catalysts, however, seems to reverse many of the demerits associated with homogeneous catalysts in the production and processing of biodiesel [258, 259]. Heterogeneous catalysts reduce energy intensity and intake as well as corrosion and toxicity, despite increasing the reaction rate time [1]. Recent developments have seen heterogeneous catalysts employed in double reaction processing for both transesterification and esterification production simultaneously. In other words, heterogeneous catalysts can catalyze in biodiesel production without an additional step of pre‐treatment to reduce FFA content [1, 260]. An increased number of new and recycled feedstock such as waste greases, and animal fats characterized with high amounts of FFA acids are becoming prominent challenging the dominance of homogeneous catalysts in the processing and production of biodiesel. Homogeneous catalysts are restricted to FFAs of a maximum of 0.5% w/w in order to avoid formation of soap and other downstream demerits previously mentioned. Soap in the transesterification causes downstream problems due to emulsification of the products in the mixture.

**FIGURE 2:**
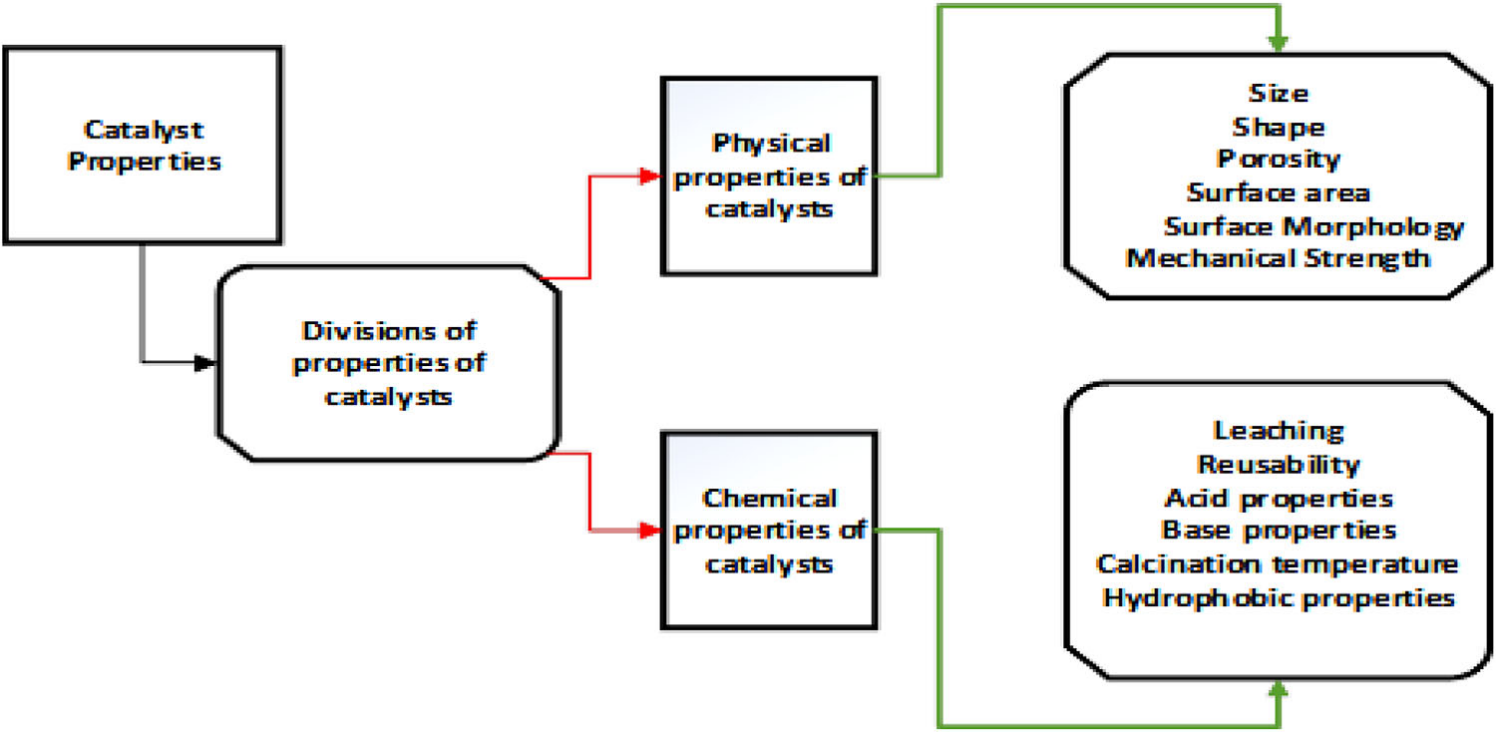
Chemical and physical properties of heterogeneous catalysts

Looking at Figure 2 it is evident that each of the physical and chemical properties of heterogeneous catalysts have the potential to influence the transesterification process [229, 261]. The literature reports additional variables, which influence heterogeneous catalyzed reactions, including the design of reactors used in production, and feedstock quality [219, 262]. This means that the quality of feedstock determines yield in respect to the life cycle analysis. In other words, feedstocks with high oil content produce high yield and vice versa. On the other hand, reactor design influences quantity of production and cost of production irrespective of catalysts used.

In the literature reviewed, a number of studies conducted experiments using alkaline earth oxides for transesterification process reactions. Examples include Ca, Mg, Sr and Ba oxides treated in high reaction temperatures (500℃ to 1050℃) [59, 263–267]. CaO particularly is reported in a number of studies to be active in refluxing methanol in vegetable oil transesterification [209, 252, 257]. These authors reported that CaO in transesterification‐catalyzed reactions are solely accelerated by nucleophilic reactions enhanced by basic properties.

The nucleophilicity of CaO which creates a dependency of susceptibility to nucleophilic attack has been reported in many studies especially for Ca(OH)_2_, CaCO_3_, MgO and Ba(OH)_2_, for example [268, 269]. MgO was found in these studies to offer more site activation at higher calcination temperatures [270]. In studies conducted by [262], SrO catalysts derived from thermal decomposition at 1200℃ give a very high yield of FAME having a long lifetime allowing reuse of up to 10 cycles and making it one of the most commercially viable catalysts likely to decrease the cost of biodiesel production.

### 5.1 Effect of base and acid catalyzed reaction on fatty acid methyl esters (FAME) yield

In the determination of catalytic activity and selectivity, the density of both the acid and base sites (read surfaces) is critical. Nevertheless, their evaluation is not easy during the transesterification process and production. Solid catalysts are known for higher rates of reactivity and faster reaction rates compared to solid acid catalysts [271]. Besides these advantages, base catalysts are very sensitive to the presence of water and FFAs. For efficient production processing, base catalysts require feedstocks with low FFAs in order to avoid catalyst deactivation [264]. On the other hand solid acid catalysts need feedstocks with high FFAs and water content but solid acid catalysts always require high operating reaction temperatures and catalyst loading to obtain reasonable biodiesel production yield (FAME) [272, 273].

Biodiesel properties depend on the different chemical and physical properties of individual fatty acid esters contained in the fuel. Therefore, the molecular structure and feedstock composition and the solvent used are critical in determining the final product. In the last two decades, considerable research has been undertaken using different catalysts aimed at obtaining specific FAME profiles and qualities [274, 275, 276]. However, in the literature surveyed the role of catalysts in the qualitative impact of catalysts is not clear [277], while the quantitative (yield) role impact of catalysts has been exhaustively discussed [211, 278]. Therefore, researchers are still faced with limited understanding in this area of catalyst behavior when under dynamic reaction operating conditions [279].

In the literature reviewed it is evident that a variety of solid acid and base heterogeneous catalysts have been studied such as alkaline earth metal oxides, hydroxides and hydrotalcites, alkali metals, hydroxides and salts supported on alumina zeolite and hydrotalcites. Table 7 is a summary of transesterification reactions using solid base catalysts for vegetable oil, the reaction operating conditions and references. From this table a number of conclusions can be drawn, for example, that KI, KF and KNO_3_ catalysts supported on alumina reported high reactivity activities at low temperatures from basic sites forming from K_2_O species by thermal decomposition or Al‐O‐K groups supported by salt support reactions [240]. Comparatively these researchers noted that Na/NaOH/γ‐Al_2_O_3_ and K/KOH/γ‐Al_2_O_3_ catalysts used during transesterification promoted strong base sites due to ionization of sodium or potassium [280]. Nevertheless the authors also noted a problem of leaching during transesterification which indicated lack of chemical stability during the reaction conditions for these group of catalysts [263]. However, Ca(NO_3_)_2_/AlO_3_ provides a high conversion rate and stability during transesterification; thus, promising its usefulness in future as a catalyst [281].

**TABLE 7:** FAME yields using acid catalysts in the transesterification of vegetable oil

Catalyst	Feedstock	Reaction operating conditions								Ref
		RT	CW	MR	TR	MFY	FP	NTY	P	
aWO_3_/ZrO_2_	Soybean	4	4	40:1	>250	0.003	0.225	90	1	[282]
aSO_4_/ZrO_2_	Soybean	4	4	40:1	300	0.002	0.200	80	1	[282]
SO_4_^2‐^/SnO_2_	Palm Kernel	4	4.01	6:1	200	0.042	0.225	90.30	50	[283]
SO_4_/ZrO_2_	Palm kernel	4	3	6:1	200	0.42	0.301	90.30	50	[283]
ZrO_2_	Palm kernel	4	2	6:1	200	0.030	0.323	64.60	50	[283]
SO_4_^2‐^/SnO_2_	Coconut	4	4	6:1	200	0.039	0.201	80.60	50	[283]
SO_4_/ZrO_2_	Coconut	4	4	6:1	200	0.042	0.215	86	50	[283]
ZrO_2_	Coconut	4	3	6:1	200	0.0243	0.123	49.20	50	[283]

CW, catalyst weight measured in grams and expressed as (NTY/FP); FP, FAME productivity (g)/t (hours) expressed as (NTY/CW); MFY, maximum FAME yield (g)/oil (g). Catalyst (g); MR, molar to oil ratio; NTY, normalized time yield is an expression of CW/FP as a percentage (CW/FP*100); P, pressure (bars); Ref, reference; RT, reaction time in (hours); TR, temperature of reaction in (℃).

### 5.2 Effect of solid catalysts in transesterification reactions

Material acidity is measured relative to base acidity or base acid interaction. In other words, in the case of Brønsted acidity the solid acid should be able to donate or transfer a proton which associates and attaches with surface anions. By definition, therefore, an acid should, as a solid acid, be able to accept an electron pair, and when reacted on acid surface should react with base molecules forming a coordinate bond. In other words, this bond described here (regarding heterogeneous acid catalysts) favors formation of electrophilic species. These species determine the rate of desorption and the rate of reaction for transesterification [284]. Nevertheless, in a study by [285] strong acid sites were noted to favor a low desorption rate which resulted in a slower process of transesterification.

### 5.3 Effect of catalyst calcination temperature on fame yield

Calcination temperature is a critical factor in the development of catalytic properties such as acid site, density, strength, molecular and crystalline structure, surface area and pore volume [66]. High calcination temperature helps to expose the catalytic sites by removing water and carbon dioxide molecules. High temperature also rearranges the bulk atoms and surface of the catalyst being produced [284].

Studies have shown that the calcination temperature affects catalyst performance. For example by calcinating CsZrO_2_/Al_2_O_3_ at 250 to 350℃ for 4 h results in an increase in FAME yield from 62 to 90% [78, 285]. However, not all increased calcination temperatures lead to increased FAME yield; excessive calcination temperature beyond a certain limit during catalyst development reduces not only FAME yield but also catalytic activity. This phenomenon is caused by gaseous diffusion through the catalyst surface pores resulting in limited surface pores or removal of binding water molecules from the catalyst structure [286].

### 5.4 Effect of reactor design and operating conditions

The reactor design and operating conditions affect exploitation of heterogeneous catalysts for biodiesel production. Globally, most commercial production of biodiesel is done through batch mode at approximately 7000 tons/year [221, 287]. However, considering the demerits of separation and the drawbacks of the batch mode, cost and capital investment, labor cost of start and stop, and increase in scale of production scale (8000‐12,500 tons/year) [221, 287, 288]. There is a need for a change to heterogeneously catalyzed continuous flow reactors for esterification of FFAs [289] such as fixed bed flow [290], micro channel flow reactors [291, 292], pervaporation techniques [293] and reactive distillation [294]. Reactive distillation in the production of biodiesel combines both the chemical and the separation stage into a single step.

**FIGURE 3:**
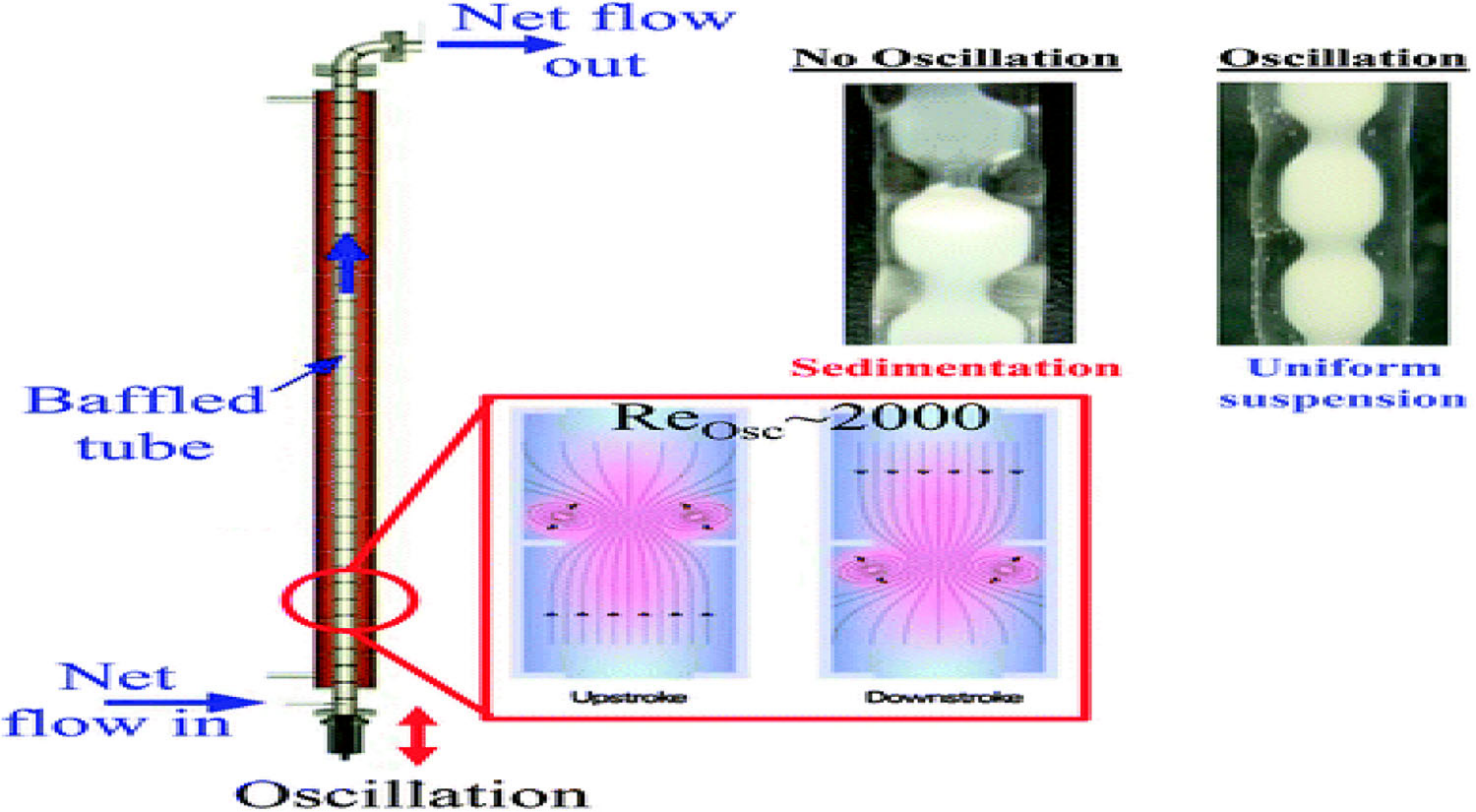
Schematic of reactor flow and mixing characteristics within an oscillatory baffled reactor, and associated optical images of a PrSO3H‐SBA‐15 solid acid powderwithout oscillation (undergoing sedimentation) or with a 4.5 Hz oscillation (entrained within baffles). Adapted from [297] with permission from The Royal Society of Chemistry

This automatically simplifies the process, reduces the cost, and extends catalyst life through continuous removal of water in the system. The reactive distillation process requires that it is compatible with temperature and pressure for distillation. Therefore, any continuous reaction reactor must utilize the full potential of control over product composition through plug flow reactors. Plug flow reactors permit tight product composition control, which in return reduces separation processes hence cost of investment and operation. Nevertheless, plug flow reactors are disadvantaged by slow reactions during esterification of FFA and TAG transesterification. This is due to the requirement for high length and diameter ratios to properly mix [295]. By introducing oscillatory baffled reactors this problem is circumvented by oscillating the reaction fluid through baffle plates to produce efficient mixing and plug flow [296]. Figure 3 shows a schematic of a set oscillatory baffled reactor showing mixing characteristics of a solid acid powder PrSO_3_H‐SBA‐15 with oscillation of a 4.5 Hz oscillation (entrained within baffles).

## 6 PRODUCTION OF BIODIESEL WITH ACID AND BASE HETEROGENEOUS CATALYSTS

Heterogeneous catalysts improve transesterification processes as these eliminate the extra costs of production and generation of pollutants [298]. Othermerits include promotion of easy product recovery, reusability and cost effective green processes. Heterogeneous catalysts are easy to recover from reaction mixtures and withstand treatment steps modified to obtain high activity, selectivity and longer catalyst life service in use [299].

In designing heterogeneous catalysts through grafting and entrapment, active molecules can be brought out of the surface from inside the pores of the supporting solid. Examples are the transesterification of FAME using a solid support such as silica alumina conan, alkali earth metals oxides, mixed metal oxides [300], transition metal oxides [301], and ion exchange resins [45]. Alkali metal compounds supported on alumina or zeolite are used in chemical reactions including isomerization, aldol condensation, knoevenagel condensation, Michael condensation, oxidation and transesterification [302].

In alkaline earth, metal oxides the successful catalysts that make the list include BeO, MgO, CaO, SrO, BaO, RaO, and MgO. Particularly important is SrO (strontium oxide) with a high basicity though it does not dissolve in methanol; thus, maintaining an efficiency of 10 cycles [219]. In other words, solid acid heterogeneous catalysts favor both esterification and transesterification of high FFA feedstocks. Solid base catalysts such as CaO, MgO, SrO, KNO_3_/Al_2_O_3_, K_2_CO_3_/Al_2_O_3_, KF/Al_2_O_3_, Li/CaO, KF/ZnO are good for transesterification [303].

In order to enhance mixed metal oxides, a number of techniques are applied such as impregnation, which was discussed extensively in Section 5. The purpose of impregnation in mixed metal oxides is to improve the base or acid strength, increase the surface area, and increase stability compared to monometal oxides [304]. For example, when MgO catalyst is doped with Li at 9%, the yield of FAME is 93.9% at low temperatures of 60℃, and a methanol to oil ratio of 12:1 [304]. Mixed metal oxide catalysts show excellent tolerance to FFA and the water content of crude oil during transesterification. Metal oxides such as CaTiO_3_, CaMnO_3_, Ca_2_FeO_5_, CaZrO_3_ and CaO‐CeO_2_ show high stability and fame yield [305].

### 6.1 Biodiesel production with mixed‐metal oxide derivatives

The use of mixed metals as catalysts in research shows high catalytic activity for the production of biodiesel, especially as supporters and promoters of active catalyst sites. This helps in reducing the amount of catalyst used in achieving a high degree of conversion and activity [306]. For example, mixed metals with sulphate metal oxides show good physical and structural properties compared to monometal oxides. Table 8 is a summary of different mixed metals and sulfated oxides used in the transesterification process and their references. The table is showing the reaction of mixed metal catalysts doped with sulfated oxides over different feedstocks summarized in the table. Different methods of catalyst preparation are used over ethanol and methanol as main solvents types. The main high point finding in this table is the high‐normalized time yields with use of mixed metal catalysts doped with sulfated oxides with an average above 90%. Among the many mixed metal oxides used in research, the mixture of CaO‐MgO is reported to be most effective [307]. In the literature surveyed, many researchers report that any addition of MgO, CaO and Zn increases catalytic activity but increases saponification [242, 308].

**TABLE 8:** Modified metal oxides and mixed‐metal oxides doped with sulfated metal oxides as solid acid catalysts for biodiesel production using esterification or transesterification reactions

Catalyst	Feed‐stock	Reaction operating conditions						Ref
		CPM	ST	MR	CW	FP	NTY	
Sr/Nio	Macaw oil	Co‐precipitation	Methanol	9:1	2	0.4850	97	[309]
Fe_2_O_3_‐SO_4_^2^^–^/SnO_2_	Lauric acid	Co‐precipitation	Methanol	–	1.5	0.5887	88.31	[310]
Fe_2_O_3_‐SO_4_^2‐^/SnO_2_/TnO	Triace‐ tin Acid	Co‐precipitation	Methanol	–	1.5	0.6000	90	[310]
Nb_2_O_5_ /SO_4_	Macaw oil	Impregnation	Methanol	120:1	5	0.1800	99	[311]
SO_4_^2–^ ‐ZnO	Soybean oil	co‐precipitation	Methanol	6:1	4	0.2000	80	[312]
(SO_4_^2‐^ /TiO_2_‐SiO_2_	Oleic acid	Sol‐gel method	Methanol	20:1	9	0.0103	92.7	[252]
	Waste oil	Sol‐gel method	Methanol	20:1	9	0.0978	88	
La_2_O_3_	Oleic acid	Impregnation	Acid: Methanol	20:1,	3	0.2667	80	[313]
Sulfated Fe_2_O_3_/TiO_2_	Vegetable oil	Sulfonation	Methanol	20:1			∼90	[314]
Mesoporous SnO_2_/WO_3_ powder	Oleic acid	–	Acid: Ethanol	120:1			∼92	[315]
SO_4_^2‐^/SnO_2_‐SiO_2_	Vegetable oil	Co‐precipitation	Methanol/Ethanol	15‐30:1			∼92	[316]

CPM, catalyst preparation method; CW, catalyst weight measured in grams and expressed as (NTY/FP); FP, FAME productivity (g)/t (hours) expressed as (NTY/CW); MR, molar to oil ratio; NTY, normalized time yield is an expression of CW/FP as a percentage (CW/FP*100); Ref, references; ST, solvent type used.

Transition metals in the literature surveyed can be modified using sulphate ions, which create acidic or superacidic catalysts [317]. The main purpose of sulfonation is improvement of surface acidity by increasing both Lewis and Bronsted acid sites, which produce electrophiles [318]. However, due to the presence of hydrophobic properties in transition metals, sulfonation is preferred for controlling hydrophobicity of metal oxides.

A number of reports published demonstrate increased solid acid heterogeneous catalysts doped with sulfated oxides. For example typical solid superacids such as SO_4_^2–^/ZrO_2_, SO_4_^2–^/TiO_2_, SO_4_^2–^/TaO_5_, SO_4_^2–^/Nb_2_O_5_ [66, 69, 263, 312–314, 316, 319, 320]. Table 9 is a summary showing modified metal oxides doped with sulfated metal oxides for the production of biodiesel. Table 9 is intended to show the interaction of modified metal oxides with doped sulfated oxides for the production of biodiesel. The table provides a summary of different feedstocks where these reactions and interaction were tested and reported in the references provided. Among the main highlights of the results summarized in this table is the average normalized time yield showing a range of 79 to 98%. This result in yield indicates very high reactivity using modified metal oxides doped with sulfated oxides.

**TABLE 9:** Summary showing modified metal oxides doped with sulfated metal oxides and their normalized time yields

Mixed sulphated metal oxide catalyst	Feedstock	Normalized time yield (%)	Reference
CaO/ZnO	Palm oil	79.60	[321]
SO_4_^2‐^/ZrO_2_	Soybean oil	80.19	[322]
SO_4_^2‐^/SnO_2_‐SiO_2_	WCO	81.40	[323]
ZrO_2_/WO_3_	Palmitic acid	98	[324]
CaO/ZnO	Palm kernel oil	>90	[325]
SO_4_^2‐^/ZrO_2_	Coconut oil	86.30	[326]
ZnO/La_2_O_3_	Rapeseed oil	86	[327]
ZrO_2_/WO_3_/Al_2_O_3_	Soybean oil	>90	[328]
CaO/Nanocatalysts	Avocado seeds	96	[329]
SO_4_^2‐^/ZrO_2_	Palm kernel oil	90.3	[326]

NTY, normalized time yield is an expression of CW/FP as a percentage (CW/FP*100).

Sulfonic acid modified catalysts can sometimes improve and produce a conversion rate of 100% FAME. However, a high concentration of methanol is detrimental to their catalytic activity [330]. Mesoporous SBA‐15 is widely used in all catalytic processes with metal oxides due to its outstanding properties such as high surface area, large uniform pore size, resistance to leaching of the supporting material and reusability capabilities [310].

### 6.2 Hydrotalcites and mixed metal oxides and derivatives in the production of biodiesel

A hydrotalcite is a double layered hydroxide with a high content of water that is used to catalyze in methanolysis of vegetable oils. Hydrotalcites (anionic clay) are naturally occurring with chemical formula in Equation 4. Where M^2+^is a di‐valent cation of either (Mg^+^, Ca^2+^, Zn^2+^, CO^2+^, Ni^2+^ or Mn^2+^). Compared to a tri‐valent M^3+^, which comprises a 3‐valent cation such as in (AL^3+^, Fe^3+^, or Cr^3+^). The layers of these elements charged positively as M^3+^ or M^2+^ cations [331–333].

(4) $$[M_{\left(1-x\right)}^{2+}M_{x}^{3+}{\left(\mathrm{OH}\right)}_{2}^{x+}.\;{\left(A_{x/\;n}^{n-}\mathrm{nH}O_{2}O\right)}^{x-}$$

Besides acting as catalysts in transesterification reactions, hydrotalcites operate as adsorbent compounds, anion exchangers [334, 335] and retardant polymers [215]. The use of hydrotalcites and mixed metals such as Mg‐Al oxide (precursors) which are homogeneously dispersed has been reported in a number of literature reviewed such as in [215, 336–338]. In all these studies, hydrotalcites and mixed metal oxides reported high catalytic activity with use of these catalysts. This is due to a strong surface basicity, high surface areas and high pore volumes similarly to the pure oxides [339, 340]. However, hydrotalcites have disadvantages such as lack of homogeneity, poor sensitivity to FFA and water in transesterification reactions [87].

In the literature surveyed many researchers report that hydrotalcites produced using alkali‐free co‐precipitation without calcination showed no catalytic activity during the transesterification process [341]. On the other hand, calcined hydrotalcites reportedly had increased catalytic activity in transesterification reaction [338, 342].

### 6.3 Performance of heterogeneous catalysts in the production of biodiesel

Many of the heterogeneous catalysts applied in the production of biodiesel are either alkali, oxide or from alkaline earth metals with support over a large surface area of precursors [265]. Solid acid catalysts are preferred compared to liquid acid catalysts due to the multiple sites strength of Lewis and Bronsted acidity [343]. Table 10 shows examples of the use of mixed metal solid acid catalysts in biodiesel production. The table provides a summary of different mixed metal catalysts reacted with different feedstocks, which are provided in the table as examples. It also provides catalyst preparation methods and the solvents used with the substrates. This table is intended to show mixed metal catalysts their preparation methods but its high point is to indicate the normalized time yields obtained with use of these types of catalysts as provided in the summary of references.

**TABLE 10:** Use of mixed metal solid acid catalysts and their preparation methods in biodiesel production

Catalyst	Feedstock	Reaction operating conditions				Ref
		CPM	ST	MR	NTY	
Cr/Al oxides	Babassu oil	Mixing	Methanol	24:1	90‐95	[308]
Fe_2_O_3_/SO_4_^2‐^/SnO_2_	Lauric acid	Homogeneously mixed	Methanol		∼79–88	[309]
Fe_2_O_3_/SO_4_^2‐^/SnO_2_/TnO	Triacetin Acid	Homogeneously mixed	Methanol		∼82–90	[309]
Nb_2_O_5_/SO_4_	Macaw oil	Impregnation	Methanol	120:1	∼99	[310]
SO_4_^2–^/ZnO	Soybean oil	Co‐precipitation	Methanol	6:1	∼75–80	[323]
SO_4_^2–^/ZnO		Impregnation				
SO_4_^2‐^/ZrO_2_	Palm kernel oil	–	20:1	6:1	∼80–90	[326]
SO_4_^2–^/SnO_2_	Crude coconut oil	–				
(SO_4_^2‐^/TiO^2‐^SiO_2_	Oleic acid	Sol‐gel method		20:1	∼93	[252]
	Waste oil		Methanol	20:1	∼88	
La_2_O_3_	Oleic acid	Impregnation	Acid: Methanol	5:1, 10:1,	∼50–99	[311]
SO_4_^2‐^/La_2_O_3_/HZSM‐5				20:1		
Nb_2_O_5_	Dodecanoic acid	Sulfonation	Acid: Alcohol	1:1–1:5	∼90	[390]
SO_4_^2‐^/ZrO_2_, SO_4_^2‐^/TiO_2_, SO_4_^2‐^/SnO_2_						
Mesoporous SnO_2_/WO_3_ powder	Oleic acid	–	Acid: Ethanol	120:1	∼92	[313]
SO_4_^2‐^/SnO_2_/Al_2_O_3_	Vegetable oil	Impregnation/sulfonation	Methanol	15:1	∼92	[392]
			Ethanol	1‐6:1	∼81	
SO_4_^2‐^/SnO_2_/SiO_2_	Vegetable oil	Co‐precipitation	Alcohol	15‐30:1	∼92	[322]

CPM, catalyst preparation method; MR, molar to oil ratio; NTY, normalized time yield is an expression of CW/FP as a percentage (CW/FP*100); Ref, references; ST, solvent type used.

#### 6.3.1 Biomass derived heterogeneous solid catalysts

Many studies have been conducted on solid alkaline and solids for the production of biodiesel. However, lower reaction rates and side reactions tend to limit wider application. This has been reported in a number of research studies [341–344]. In the last decade solid alkaline for biodiesel production has seen tremendous development. For example, the widespread use of calcium oxide obtained from biomass sources and conventional sources. CaO provides a number of advantages such a high activity, long life during use in moderate conditions for reactions. Nevertheless, it has been reported to have lower reaction rates compared to other heterogeneous catalysts [273].

A number of researchers have used CaO as a solid alkaline catalyst for biodiesel production for example [229, 277, 345–348]. The literature reviewed report high solubility of CaO in methanol; thus, making it an attractive recyclable candidate for catalysts [76, 349]. Other studies have combined mixed oxide heterogeneous catalysts such as calcium doped with silicate to produce biodiesel from soybean and other feedstocks. Examples of silicate containing mixed calcium oxide catalysts include PME template calcium (PMCS1‐9) and mesoporous calcium CS 1–9 [350, 351].

### 6.4 Zeolite based catalysts in the production of biodiesel

Zeolite catalysts are microporous crystalline alumina silicate compounds composed of a three dimensional porous structure consisting of TO_4_ tetrahedra connected with O atoms [352]. Zeolites allow certain hydrocarbon molecules to enter their pores while at the same time rejecting others. The rejection of these others is based on too large molecular size, the ion exchange properties or the ability to develop internal acidity [85, 353]. The commercial interest in zeolites follows decades of research in the 1970s building on research discoveries of the 1950s. This has revolutionized the petrochemical industry due to the number of applications offered by zeolites. For example, the use of zeolites in catalytic cracking, alkylation, and isomerization [354].

Although zeolites are active catalysts for carboxylic acid during esterification, their reaction rate is slow as only large pore zeolites are successfully used. Zeolites catalytic activity is greatly influenced by site strength and surface hydrophobicity pore size, dimensionality of the channel system and the aluminum content [320].

The advantages zeolites include a combination of several key properties such as high surface area, good ion exchange ability, high acidity, high thermal ability and hydrothermal stability [355, 356]. Technical advantages of zeolites include shape discrimination of reactants, products and transition states. Zeolites can be made for specific acid‐base catalyzed reactions and synthesized using various crystal structures, pore sizes framework of Si/Al ratios and proton exchange levels. All these occur in a molecular state due to the regular crystalline channels, which help to form shape selective reactions [356]. A number of researchers such as [278, 357–361] have reported research.

In the last two decades, a lot of research has focused on accessibility of active sites in microporous zeolite frameworks. As a result, two strategies to obtain hierarchical structure in zeolites namely the “top‐down” and the “bottom‐up” came into existence [362]. The top‐down approach starts with microporous zeolites modified synthetically to create a hierarchical structure, for example, the pioneering work of dealumination [363]. The top‐down approach focuses on ensuring optimization of the dealumination of zeolites in order to obtain an ultra‐stable form where the Si/Al ratio is higher for the purposes of catalytic cracking [364, 365]. On the other hand, the bottom up approach uses engineering of microporous and mesoporous domains to build hierarchical zeolites with template materials. Most often carbon materials are used as template materials due to their versatility and availability. For example, carbon black, carbon nanotubes and nanotubes, carbon aerogel and other mesoporous carbon materials [366, 367].

Using carbon templates in a bottom‐up approach ensures production of a wider variation of mesoporous structures without interconnection but highly ordered and networked during the synthesis process. This is demonstrated in a number of research projects although it is more difficult with graphene and graphene oxide template material [368]. Table 11 shows zeolite based heterogeneous catalysts commonly used and their sample of reaction operating conditions for biodiesel production. The table highlights the use of different use of different group of heterogeneous catalysts that are zeolite based. The table also shows different feedstock sources reacted with the catalysts and the reaction temperatures during experimentation. The table describes in length and summarizes the reaction operating conditions and calculation for normalized time yield as a function of catalyst weight and FAME. Among the highlight of the result using zeolite‐based catalysts as reported in this table as summarized from the references provided indicates a yields range of 79.30 to 98%.

**TABLE 11:** Heterogeneous catalysts commonly used for biodiesel production

Catalyst	Source	Reaction operating conditions						Ref
		TR	RT	MR	CW	FP	NTY	
Mg‐Al hydrotalcite	Jatropha	45	1.5	4:1	1	0.95	95	[369]
K_2_CO_3_ supported MgO	Soybean	70	2	6:1	0.7	1.4	98	[370]
Mg/Zr	Sunflower	65	50 min	53:1	0.1	9.8	98	[371]
Fe‐Zn Double metal cyanide (DMC) complex	Sunflower	170	8	15:1	3	0.32	96	[372]
SO_4_^2^/TiO_2_	Jatropha	90	3	20:1	4	0.24	96	[273]
ZS/Si Waste	Cooking oil	200	10	1:1	0.2	4.9	98	[374]
Vanadium phosphate solid	Soybean	150	1	1:1	0.2	4	80	[375]
Al_2_O_3_/ZrO_2_/WO_3_	Soybean	200	6	32:1	1	0.9	90	[376]
SBA‐15‐SO_3_H‐P123 Solphonic acid supported on mesoporous silica	Soybean	75	20	20:1	10	0.085	85	[377]

CW, catalyst weight measured in grams and expressed as (NTY/FP); FP, FAME productivity (g)/t (hours) expressed as (NTY/CW); MR, molar to oil ratio; NTY, normalized time yield is an expression of CW/FP as a percentage (CW/FP*100); Ref, references; RT, reaction time in (hours); TR, temperature of reaction in (℃).

### 6.5 Microbubble mediated catalysis as a heterogeneous biodiesel production method

Although it a novel method in the area of catalysts its predecessors such as reactive distillation (RD), pressure swing reactive distillation (PSRD) have been used in a number reported experiments. This method increases the reaction rate and conversion compared to conventional esterification where kinetic and mass transfer present challenges [378]. This method of catalysis has been developed as a response to the challenges in esterification using the methods described and discussed in this section (6). Microbubbles reactive method of catalysis produces less buoyancy, hence increasing residence time in the liquid bulk [379–382]. A number of researchers have reported work using this method as a form of heterogeneous catalyst under reactive distillation such as [378, 379, 383]. However, since it is a new area these articles will not review this method here at length but in future reviews this method will feature as more researchers report findings.

## 7 THE SWOT ANALYSIS OF BIOMASS DERIVED HETEROGENEOUS CATALYSTS

There are a number of problems faced by solid catalysts such as the low number of active sites, micro porosity, leaching, toxicity, and expense [24]. This arises from the fact that many homogeneous catalysts are derived from non‐renewable resources, which are environmentally unfriendly. The three phase heterogeneous system diffuses this problem through reaction inhibition [1]. It should be noted that the three phases of solid catalysts make them highly immiscible; thus, limiting their mass transfer efficiency hence lower reaction rates [23]. The inhibition of the mass transfer capabilities of larger molecules results in poor conversion yield rates for biodiesel [384]. Therefore, to produce excellent solid acid catalysts, the catalyst must have more specific surface areas (hydrophobicity, external catalytic sites, etc.) and a large pore diameter [23].

Exploration of feedstocks and biomass catalysts especially from waste biomass sources and materials should encouraged. The process needs affordability and sustainability while reducing environmental degradation. More resources committed to research on performance of catalysts is needed in order to increase awareness of the application of waste to create sustainability. Through financial and tax incentives, coupled with regulations to allow use of recycled materials in production of new products, the goal can be achieved of creating a circular economy with producer and consumer responsibility at the core of the business plan.

Globally we are witnessing a steady growth in energy demand especially in the transport sector which accounts for 40.5% of all global energy consumption [10]. There is an urgent need to reduce heavy dependency on fossil fuels, by developing alternative energy sources such as biofuels and biodiesel. This requires sound and equitable use of natural resources such as vegetation, biomass and bio‐waste and microorganisms to meet this growing energy demand for alternative energy sustainably.

Biodiesel processing and production globally has witnessed an increased growth both in use and in production but with homogeneous catalysts being used most commonly. This has led to poor utilization of all potential biodiesel feedstocks available, as there are many demerits related to production. Therefore, the novel introduction of heterogeneous catalysts was a timely development to counter the demerits of homogeneous technologies in production of biodiesel. Heterogeneous catalysts simplify production by reducing by‐products such as soap, by increasing catalyst reuse, by faster production of biodiesel and by being ecofriendly and sustainable. However, heterogeneous catalysts fail to produce biodiesel that meet FAME content standards (>96.5%) [265]. Therefore, in future, there is a need to upgrade production platforms through process variability and catalyst tuning by modification to improve reaction performance [265].

In other words although heterogeneous catalysts have shown greater potential in biodiesel production there are gaps in the literature surveyed regarding data on kinematics and reaction mechanisms especially during transesterification and esterification processes [385]. The literature available is insufficient to draw conclusions on mechanisms and kinematics of heterogeneous catalysts [54, 386]. This calls for more and extensive research on heterogeneous catalysts given that there is a disproportionate study on the demerits and merits.

The challenges of homogeneous catalyst have been exhaustively studied in the literature reviewed, and some of which have been addressed. Heterogeneous catalysts are a new research area with significant ongoing research. Several challenges have been reported in the literature for these catalysts and need attention, such as short catalyst life, low reaction rate, and instability. The solid base catalysts reviewed in the literature show sensitivity to CO_2_, water, and FFAs. Therefore, increasing the consumption and deactivation of catalysts via saponification.

Other reported challenges include induced catalyst leaching and final product contamination due to hydrolization of the ionic group by water, lipase inhibition in the presence of methanol during enzymatic biodiesel synthesis processes. In the case of nanocatalysts, it is necessary to increase reaction temperatures to achieve high performance in mild operating conditions, which increases cost and energy requirements. Therefore, energy efficient and low cost methods are needed to develop nanocatalysts for effective recovery and reuse of these catalysts (Tables 12 and 13).

**TABLE 12:** Advantages of biomass derived heterogeneous catalysts

Advantages (strengths)	Reference
High availability and reactivity	[25, 27, 31]
Derived from waste and end product industrial processes hence low cost and eco‐friendly	[74, 75, 77, 78]
Have the ability to perform both esterification and transesterification processes for the production of biodiesel	[84]
Simplifies production processes, low energy consumption, high product purity of glycerol, less soap formation during reaction, easy separation of catalyst from reaction mixture	[6]
Allows reuse of immobilized enzymes by the process of immobilization through a selection process of genetic engineering	[383]
Use of mild reaction temperatures	[384]
High selectivity and specificity of transesterification towards substrates, eliminates treatment costs associated with chemical catalysts recovery, environmental friendly and biodegradable	[385]

**TABLE 13:** Disadvantages of biomass derived heterogeneous catalysts

Disadvantages (weaknesses)	References
Enzyme catalysts are sensitive to water making it harder to use polar substrates such as water, methanol and glycerol and phospholipids.	[10]
Low reactivity rate for FFAs at <1 wt% and enzyme inhibition	[88]
Saponification as a side effect reaction and soap formation, high volumes of wastewater formation, leaching of catalyst sites, limitation in diffusion, complex and expensive synthesis routes, high costs of catalysts, highly sensitive to alcohol, denaturation of enzymes	[1, 19]

### 7.1 Opportunities for biomass derived catalysts

Many novel ideas such as use of solid whole cell biocatalysts using solid‐state fermentation have been developed. This method allows low microorganism growth on the substrate (agro‐industrial waste residue). Second, it allows crude fermented solid material to be directly used as biocatalysts thereby reducing lipase purification and immobilization steps [6]. The other opportunities available for use of biocatalytic material are the use of hydro‐esterification, which was recently developed. This was in response to feedstocks with a high percentage of FFAs and water. This process occurs into main stages where in stage one only mono and triacylglycerols are hydrolyzed to FFA and glycerol. On the other hand, stage two involves separation of the FFAs and esterified biodiesel [385].

Under current research for methanol inhibition which is a common occurrence with the use of enzymes. Researchers recommend the use of solvent and continuous removal of glycerol by dialysis or solvent extraction or a stepwise addition of methanol. However, the current trend is the use of the recombinant DNA technology to address this problem [386]. On the other hand, in order to reduce reaction time and minimize catalyst inhibition. There has been a development a continuous system based on near critical carbon dioxide as the reaction medium [337]. Another area, which offers greater opportunity in the catalytic processes, is the production of syngas from glycerol a by‐product of esterification. This involves auto‐thermal reforming aqueous phase reforming and supercritical water reforming [387, 388, 389].

Despite the current high prices of lipase methyl and ethyl acetate which are used as acyl accepters are becoming substitutes for alcohols [272]. This technique ensures that no glycerol is formed hence eliminating the down steam separation and recovery of glycerol. The final product is a new product of higher value called triacetin hence low cost of production.

### 7.2 Future recommendations

The following aspects need to be addressed in future works: (1) Further studies on waste derived catalysts in order to develop improved new catalysts for the synthesis of biodiesel. (2) Development of a highly active and selective heterogeneous catalyst enabling commercialization of heterogeneous catalysts going forward. In other words, there has to be improvement and development in terms of performance in biomass‐derived catalysts for biodiesel production and other essential chemical processes. This catalyst should be able to provide and support interconnected systems of appropriate pores sizes within catalysts. As more research is advanced to improve heterogeneous catalysts from waste biomass the cost and sustainability of catalyst will be secured in order to transform into commercialization of solid catalysts. (3) Improve preparation of hydrotalcite and zeolite based catalysts to be used in industrial and commercial scale. This can be achieved by improving their sensitivity to FFA and water by improving the basicity of these catalysts. (4) In the literature reviewed CaO based catalysts reported high reactivity compared to other catalysts due to long time use and high activity in mild reaction operating conditions. Therefore, time and effort should be directed in improving performance in the area of kinetics rate of reaction. (5) In the literature surveyed the role of the qualitative impact of catalysts is not clear while the quantitative (yield) impact of catalysts has been exhaustively discussed. Therefore, researchers are still faced with limited understanding in this area of catalyst behavior when under dynamic reaction operating conditions [279]. For further discussion of this point, the reader can refer to the reference provided here. (6) In developing waste to catalysts there is need for government support through tax reduction or relief and introduction of favorable regulation, which encourage use of recycled products from waste. In other words, if supported waste derived catalysts have a potential of job creation and support industry.

## CONFLICT OF INTEREST

The authors have declared no conflict of interest.

## Data Availability

Data sharing is not applicable to this article as no new data were created or analyzed in this study.
